# Dataset on experimental investigation of gum arabic coated alumina nanoparticles for enhanced recovery of nigerian medium crude oil

**DOI:** 10.1016/j.dib.2018.05.046

**Published:** 2018-05-23

**Authors:** Oyinkepreye D. Orodu, Kale B. Orodu, Richard O. Afolabi, Eboh A. Dafe

**Affiliations:** Department of Petroleum Engineering, Covenant University, P.M.B 1023, Ota, Ogun State, Nigeria

**Keywords:** Oil recovery, Waterflooding, Gum arabic, Nanoparticles, Nigerian medium crude oil

## Abstract

The dataset in this article are related to an experimental Enhanced Oil Recovery (EOR) scheme involving the use of dispersions containing Gum Arabic coated Alumina Nanoparticles (GCNPs) for Nigerian medium crude oil. The result contained in the dataset showed a 7.18% (5 wt% GCNPs), 7.81% (5 wt% GCNPs), and 5.61% (3 wt% GCNPs) improvement in the recovery oil beyond the water flooding stage for core samples A, B, and C respectively. Also, the improvement in recovery of the medium crude oil by the GCNPs dispersions when compared to Gum Arabic polymer flooding was evident in the dataset.

**Specifications Table**

**Table:** 

Subject area	*Petroleum Engineering*
More specific subject area	*Enhanced Oil Recovery/Tertiary Oil Recovery*
Type of Data	*Tables and Figures*
How Data was Acquired	*Core Flooding Experiment using the OFITE*^*®*^*Reservoir Permeability Tester*
Data Format	*Raw Data*
Experimental Factors	1. *GCNP preparation using Al*_*2*_*O*_*3*_ *nanoparticles and Gum Arabic* 2. *Core plugs were cleaned with acetone using the Soxhlet apparatus* 3. *Saturation of the plugs were done using Vinci Technologies*^*®*^ *High Pressure Core Saturator* 4. *Core flooding of the plugs using OFITE*^*®*^ *Reservoir Permeability Tester at different flow rates for waterflood and GCNP*
Experimental Features	*Improvement in recovery of the medium crude oil by the GCNPs dispersions when compared to water or Gum Arabic polymer flooding*
Data Source Location	*Department of Petroleum Engineering, Covenant University, Nigeria*
Data Accessibility	*Data is with the article*

**Value of data**

• Core flooding results show the relevance of polymer coated nanoparticles for the recovery of crude oil from conventional reservoirs.
• The GCNPs provided improved recovery of oil beyond the capacity of water flooding and polymer flooding.
• Incremental oil recovery over that of waterflooding was encouraging despite permeability impairment by about half the initial measured value.
• The results obtained calls for a detailed study on the mechanisms at play with respect to the polymeric and surfactant property of Gum Arabic. Likewise, the performance of Gum Arabic should be evaluated and compared to that of known and standard polymers used in the industry.

## 1 Data

**Fig. 1:**
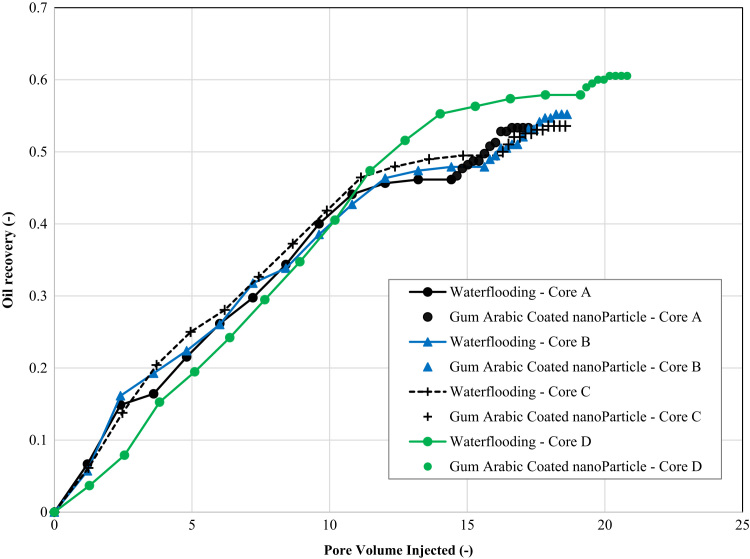
Effect of GCNPs on the EOR process after water flooding for cores A, B, C and D.

Nanoparticles are reported in [1], [2], [3] to improve oil recovery but its instability paved the way for stable polymer coated nanoparticles [4]. The dataset presented in this paper provides an experimental investigation of Gum Arabic coated Alumina Nanoparticles (GCNPs) for enhanced recovery of Nigerian medium crude oils. Gum Arabic is a naturally occurring polymer that is abundant in Nigeria and Sudan. Table 1 shows the properties of the various cores, inclusive of the impact of GCNPs flooding on permeability causing impairment of the cores. Table 2 shows the results for the determination of connate water saturation in the cores after the oil injection process. Table 3 gives values for the residual oil saturation and recovery factors after water flooding. Table 4 gives the additional oil recovery obtained using GCNPs and the irreducible oil saturation. Whereas Fig. 1 displays graphically, the impact of the incremental oil recovered by GCNPs after the optimal recovery by the waterflooding process. The dataset for Fig. 1 is presented in Table 5.

**Table 1:** Rock properties of the Berea cores. The effect of the GCNPs on the absolute permeability are captured in the last two columns.

**Core samples**	**Length**	**Diameter**	**Bulk volume**	**Wet weight**	**Dry weight**	**Pore volume**	**Porosity**	**Absolute K (Pre flooding)**	**Absolute K (Post flooding)**
	(cm)	(cm)	(ml)	(g)	(g)	(ml)	(%)	(mD)	(mD)
Core A	6.30	3.7	67.77	165.3	151.2	12.48	18.41%	262.3	125.8
Core B	6.25	3.7	67.23	165.1	151.0	12.48	18.56%	278.8	115.4
Core C	6.30	3.7	67.77	164.7	151.0	12.12	17.89%	251.7	173.2
Core D	6.25	3.7	67.23	165.2	151.9	11.77	17.51%	245.0	223.7

**Table 2:** Determination of connate water saturation from oil injection process.

**Core**	**Total pore volume of the core (ml)**	**Volume of water expelled from core (ml)**	**Total oil in place (ml)**	**Connate volume of water (ml)**	$S_{\mathrm{oi}}$	$S_{\mathrm{wc}}$
A	12.48	9.75	9.75	2.73	0.78	0.22
B	12.48	9.60	9.60	2.88	0.77	0.23
C	12.12	9.80	9.80	2.32	0.81	0.19
D	11.77	9.50	9.50	2.27	0.81	0.19

**Table 3:** Residual oil saturation and recovery factor after water flooding process.

**Cores**	**Total recovered oil volume**	**Residual oil volume**	$S_{w}$	$S_{\mathrm{or}}$	**Recovery factor**
	mL	mL			%
A	4.50	5.25	0.58	0.42	46.15%
B	4.55	5.05	0.60	0.40	47.40%
C	4.70	5.10	0.58	0.42	47.96%
D	5.50	4.00	0.66	0.34	57.89%

**Table 4:** Additional oil recovery using GCNPs and the irreducible oil saturation.

**Cores**	**Total recovered oil volume**	**Residual oil volume**	$S_{\mathrm{oirr}}$	**Additional recovery**	**Recovery factor**
	mL	mL		%	%
A	5.20	4.55	0.36	7.18%	53.33%
B	5.30	4.30	0.34	7.81%	55.21%
C	5.25	4.55	0.38	5.61%	53.57%
D	5.75	3.75	0.32	2.63%	60.53%

**Table 5:** Oil recovery of GCNPs assisted waterflooding for cores A, B, C and D.

Core A (GCNPs 5 wt%)			Core B (GCNPs 5 wt%)			Core C (GCNPs 3 wt%)			Core D(GCNPs 3 wt%)		
Flooding Rate	Pore Volume Injected	Oil Recovery	Flooding Rate	Pore Volume Injected	Oil Recovery	Flooding Rate	Pore Volume Injected	Oil Recovery	Flooding Rate	Pore Volume Injected	Oil Recovery
(cc/min)	(-)	(-)	(cc/min)	(-)	(-)	(cc/min)	(-)	(-)	(cc/min)	(-)	(-)
H2O 3cc/min	0	0	H2O 3cc/min	0	0	H2O 3cc/min	0	0	H2O 3cc/min	0	0
H2O 3cc/min	1.201923	0.066667	H2O 3cc/min	1.201923	0.057292	H2O 3cc/min	1.237624	0.061224	H2O 3cc/min	1.274427	0.036842
H2O 3cc/min	2.403846	0.148718	H2O 3cc/min	2.403846	0.161458	H2O 3cc/min	2.475248	0.137755	H2O 3cc/min	2.548853	0.078947
H2O 3cc/min	3.605769	0.164103	H2O 3cc/min	3.605769	0.192708	H2O 3cc/min	3.712871	0.204082	H2O 3cc/min	3.82328	0.152632
H2O 3cc/min	4.807692	0.215385	H2O 3cc/min	4.807692	0.223958	H2O 3cc/min	4.950495	0.25	H2O 3cc/min	5.097706	0.194737
H2O 3cc/min	6.009615	0.261538	H2O 3cc/min	6.009615	0.260417	H2O 3cc/min	6.188119	0.280612	H2O 3cc/min	6.372133	0.242105
H2O 3cc/min	7.211538	0.297436	H2O 3cc/min	7.211538	0.317708	H2O 3cc/min	7.425743	0.326531	H2O 3cc/min	7.646559	0.294737
H2O 3cc/min	8.413462	0.34359	H2O 3cc/min	8.413462	0.338542	H2O 3cc/min	8.663366	0.372449	H2O 3cc/min	8.920986	0.347368
H2O 3cc/min	9.615385	0.4	H2O 3cc/min	9.615385	0.385417	H2O 3cc/min	9.90099	0.418367	H2O 3cc/min	10.19541	0.405263
H2O 3cc/min	10.81731	0.441026	H2O 3cc/min	10.81731	0.427083	H2O 3cc/min	11.13861	0.464286	H2O 3cc/min	11.46984	0.473684
H2O 3cc/min	12.01923	0.45641	H2O 3cc/min	12.01923	0.463542	H2O 3cc/min	12.37624	0.479592	H2O 3cc/min	12.74427	0.515789
H2O 3cc/min	13.22115	0.461538	H2O 3cc/min	13.22115	0.473958	H2O 3cc/min	13.61386	0.489796	H2O 3cc/min	14.01869	0.552632
H2O 3cc/min	14.42308	0.461538	H2O 3cc/min	14.42308	0.479167	H2O 3cc/min	14.85149	0.494898	H2O 3cc/min	15.29312	0.563158
GCNP 0.5cc/min	14.6234	0.466667	H2O 3cc/min	15.625	0.479167	H2O 3cc/min	16.08911	0.494898	H2O 3cc/min	16.56754	0.573684
GCNP 0.5cc/min	14.82372	0.476923	GCNP 0.5cc/min	15.82532	0.489583	GCNP 0.5cc/min	16.29538	0.5	H2O 3cc/min	17.84197	0.578947
GCNP 0.5cc/min	15.02404	0.482051	GCNP 0.5cc/min	16.02564	0.494792	GCNP 0.5cc/min	16.50165	0.510204	H2O 3cc/min	19.1164	0.578947
GCNP 0.5cc/min	15.22436	0.487179	GCNP 0.5cc/min	16.22596	0.505208	GCNP 0.5cc/min	16.70792	0.520408	GCNP 0.5cc/min	19.3288	0.589474
GCNP 0.5cc/min	15.42468	0.487179	GCNP 0.5cc/min	16.42628	0.505208	GCNP 0.5cc/min	16.91419	0.520408	GCNP 0.5cc/min	19.54121	0.594737
GCNP 0.5cc/min	15.625	0.497436	GCNP 0.5cc/min	16.6266	0.510417	GCNP 0.5cc/min	17.12046	0.52551	GCNP 0.5cc/min	19.75361	0.6
GCNP 0.5cc/min	15.82532	0.5078	GCNP 0.5cc/min	16.82692	0.510417	GCNP 0.5cc/min	17.32673	0.52551	GCNP 0.5cc/min	19.96602	0.6
GCNP 0.5cc/min	16.02564	0.512821	GCNP 0.5cc/min	17.02724	0.520833	GCNP 0.5cc/min	17.533	0.530612	GCNP 0.5cc/min	20.17842	0.605263
GCNP 0.5cc/min	16.22596	0.528205	GCNP 0.5cc/min	17.22756	0.53125	GCNP 0.5cc/min	17.73927	0.530612	GCNP 0.5cc/min	20.39082	0.605263
GCNP 0.5cc/min	16.42628	0.528205	GCNP 0.5cc/min	17.42788	0.53125	GCNP 0.5cc/min	17.94554	0.535714	GCNP 0.5cc/min	20.60323	0.605263
GCNP 0.5cc/min	16.6266	0.533333	GCNP 0.5cc/min	17.62821	0.541667	GCNP 0.5cc/min	18.15182	0.535714	GCNP 0.5cc/min	20.81563	0.605263
GCNP 0.5cc/min	16.82692	0.533333	GCNP 0.5cc/min	17.82853	0.546875	GCNP 0.5cc/min	18.35809	0.535714			
GCNP 0.5cc/min	17.02724	0.533333	GCNP 0.5cc/min	18.02885	0.546875	GCNP 0.5cc/min	18.56436	0.535714			
GCNP 0.5cc/min	17.22756	0.533333	GCNP 0.5cc/min	18.22917	0.552083						
GCNP 0.5cc/min	17.42788		GCNP 0.5cc/min	18.42949	0.552083						
			GCNP 0.5cc/min	18.62981	0.552083						

## 2 Experimental design, materials and methods

### 2.1 Core cleaning

The Berea sandstone cores (labelled A, B, C and D, all purchased from Cleveland Quarries Inc.) were immersed in acetone vapors (at 110 °C), as acetone (analytical grade) is boiled slowly in a Pyrex flask with its vapor moving upwards in a Soxhlet apparatus. Water contained in the thimble housing the core sample in the thimble is vaporized. Re-condensed acetone together with liquid water falls from the base of the condenser onto the core sample in the thimble; the acetone soaks the core sample and dissolves any oil with which it comes into contact. When the liquid level within the Soxhlet tube reaches the top of the siphon tube arrangement, the liquids within the Soxhlet tube are automatically emptied by a siphon effect and flow into the boiling flask. The acetone is then ready to start another. Afterwards, a desiccator was employed in drying the core samples.

### 2.2 Preparation of brine

The brine was prepared to about 3.0 wt.% (0.03 g/ml). 30 g of NaCl salt (analytical grade) was measured with the use of the weighing balance and diluted in 750 ml of water. The salt was poured into the cylinder and stirred properly so as to dissolve evenly. Then water was poured into the measuring cylinder filling it up to 1000 ml.

### 2.3 Preparation of gum arabic coated nanoparticles (GCNPs)

The nanoparticle in use was Al_2_O_3_ (30–60 nm, purity greater than 99%; manufactured by Sigma Aldrich and purchased from Equilab Solutions in Nigeria.). 50 g of Al_2_O_3_ was dispersed in 1 l of deionized water to make nano-fluid suspensions, making a 5 wt.% mixture. It was further diluted to 3 wt% in order to completely carry out further experiments. The Gum Arabic (a polymer; purchased locally in Nigeria) was mixed with the prepared nanofluids at a concentration of 10 wt.%.

### 2.4 Determination of porosity and absolute permeability

The dimensions of the cleaned dry cores (length, diameter and weight) were taken before being saturated with brine using the Vinci Technologies® High Pressure Core Saturator. The pore volume for each core was calculated as;

**Table:** 

Length of core = $L_{c}$	Diameter of core = $D_{c}$ (radius = $r_{c}$)
Bulk volume of core = $V_{T}$ = $\pi r_{c}^{2}L_{c}$	Weight of dry core = $W_{D}$
Weight of core saturated with brine = $W_{s}$	Density of brine = $\rho_{b}$ = 1.13 g/cm^3^
Pore volume = $V_{p}$ = $\frac{W_{s}-W_{D}}{\rho_{b}}$	Porosity = $\emptyset =\frac{V_{p}}{V_{T}}$

The permeability of the cores was determined using the reservoir permeability tester.

### 2.5 Core flooding

The cores were saturated with 100% brine and the flooding experiments started with a primary drainage process. Oil was injected into the core plugs at 5 cc/min until brine was no longer produced. This procedure established the initial/connate water saturation, ‘$S_{\mathrm{wc}}$’. The next stage was the water flooding; water was injected into the core plugs at 3 cc/min until oil was no longer produced for secondary recovery. This established the residual oil saturation, ‘$S_{\mathrm{or}}$’. GCNPs and polymers were initiated as an enhanced oil recovery (EOR) process. To investigate if they had any effect on the oil recovery, “they were injected into the core plug after the water flooding”. The extra oil produced during the EOR process increased the recovery factor and hence proved that GCNPs potentially can work as an EOR agent (Fig. 1). As there was no automated way to measure the recovery, the experiment had to be monitored during the whole flooding sequence. Samples of the effluent fluids were manually taken every (five) 5 min at the outlet of the core holder in test tubes. The samples were used to measure the amount of oil and brine produced and used for calculating saturations as well as recovery factor.
